# The association of vitamin D deficiency with the severity of adenoid hypertrophy and allergic rhinitis in children: a retrospective observational study

**DOI:** 10.3389/fnut.2026.1793848

**Published:** 2026-04-13

**Authors:** Yating You, Ying Wang, Jian Zhu, Rui Guo

**Affiliations:** Department of Otaryngology, Head and Neck Surgery, Changde Hospital, Xiangya School of Medicine, Central South University (The First People’s Hospital of Changde City), Changde, Hunan, China

**Keywords:** adenoid hypertrophy, allergic rhinitis, children, immunomodulation, vitamin D deficiency

## Abstract

**Background:**

Allergic rhinitis (AR) and adenoid hypertrophy (AH) are common pediatric comorbidities. Vitamin D is a known immunomodulator, but its relationship with the concurrent severity of AR and AH remains unclear. We aimed to investigate the association between serum 25-hydroxyvitamin D [25(OH)D] levels and disease severity in children with both conditions.

**Methods:**

This retrospective study included 268 children (3–12 years) with co-existing AR and AH. We collected data on serum 25(OH)D, adenoidal-nasopharyngeal (A/N) ratio, total and specific IgE, and symptom scores. Patients were stratified by 25(OH)D levels: severe deficiency (<10), deficiency (10–<20), insufficiency (20–<30), and sufficiency (≥30 ng/mL). Multiple linear/logistic regression and restricted cubic spline (RCS) analyses were performed.

**Results:**

The mean 25(OH)D level was 17.7 ± 6.8 ng/mL, with a high prevalence of deficiency (70.5% < 20 ng/mL). Serum 25(OH)D levels were significantly and inversely correlated with A/N ratio (*r* = −0.34), total IgE (*r* = −0.34), and symptom scores (*r* = −0.50). After full adjustment, each 10 ng/mL decrease in 25(OH)D was associated with a 0.055-unit increase in A/N ratio. Compared to sufficiency, severe deficiency conferred a 7.13-fold increased risk of severe AH (A/N ratio ≥0.80) and a 7.83-fold increased risk of high total IgE (>400 kU/L). RCS analysis revealed a significant nonlinear dose–response relationship, with the strongest association observed at 25(OH)D levels below 20–25 ng/mL.

**Conclusion:**

Vitamin D deficiency is highly prevalent and independently associated with increased severity of both adenoid hypertrophy and allergic inflammation in children with AR and AH. This association is most pronounced at lower vitamin D concentrations, suggesting that vitamin D status may play a role in the pathophysiology of these conditions and that correcting deficiency could be a valuable adjuvant therapeutic strategy.

## 1 Introduction

Allergic rhinitis (AR) and adenoid hypertrophy (AH) are two of the most prevalent and frequently co-existing chronic conditions in pediatric medicine, constituting a significant public health burden globally. AR, characterized by an IgE-mediated inflammation of the nasal mucosa, affects up to 35% of 13–14-year-olds, while AH, the enlargement of the nasopharyngeal lymphoid tissue, is a primary cause of nasal obstruction and sleep-disordered breathing (SDB) in children (1). The interplay between these two conditions is widely recognized; the persistent inflammation of AR can promote adenoid hyperplasia, and in turn, enlarged adenoids can exacerbate nasal congestion and related symptoms (2). This vicious cycle severely impairs a child’s quality of life, leading to disturbed sleep, daytime fatigue, and significant long-term sequelae, most notably the development of “adenoid facies”—a constellation of craniofacial alterations resulting from chronic mouth breathing. Given that AH is the most common cause of pediatric obstructive sleep apnea (OSA) (3), and current management strategies, including pharmacotherapy, adenoidectomy, and allergen-specific immunotherapy, have notable limitations and controversies, there is a pressing need for a deeper understanding of the modifiable risk factors that underpin their shared pathophysiology (3).

In recent years, the potential role of vitamin D has expanded far beyond its classical function in calcium homeostasis. It is now firmly established as an important immunomodulator, and its levels are linked to profound effects on both the innate and adaptive immune systems (4). The vitamin D receptor (VDR) is expressed by the majority of immune cells, including B and T lymphocytes, monocytes, macrophages, and dendritic cells (5). Through VDR activation, the active form of vitamin D, 1,25-dihydroxyvitamin D3 [1,25(OH)2D3], orchestrates a complex network of immune responses. It enhances innate immunity by promoting the production of antimicrobial peptides such as cathelicidin, while concurrently shaping adaptive immunity by suppressing the pro-inflammatory Th1 phenotype and, crucially for allergic diseases, downregulating the Th2-driven immune response (6). This is achieved by downregulating the levels of key Th2 cytokines like interleukin-4 (IL-4) and IL-5, which are central to IgE production and eosinophil recruitment. Furthermore, vitamin D promotes immune tolerance by fostering the development and function of regulatory T cells (Tregs) and is associated with regulatory B cell (Breg) populations that produce the anti-inflammatory cytokine IL-10 (5, 7).

This immunoregulatory capacity has positioned vitamin D as a key environmental factor in the pathogenesis of allergic diseases. A growing body of evidence, including meta-analyses, has demonstrated a significant association between low serum 25(OH)D levels and an increased risk of AR in children (8). Observational studies suggest that vitamin D deficiency is more prevalent in AR sufferers (9), and some, though not all, intervention trials indicate that supplementation may alleviate symptoms (10).

Concurrently, a parallel line of inquiry has emerged linking vitamin D status to AH. Several studies have reported that children with AH and related complications like OSA have significantly lower serum 25(OH)D levels compared to healthy controls, with one study showing mean plasma 25hydroxy vitamin D levels of 17 ng/mL versus 22 ng/mL in controls (11) and another finding mean serum 25(OH)D levels of 18.4 ng/mL in children with adenotonsillar hypertrophy versus 22.5 ng/mL in controls (12). This has led to the hypothesis that vitamin D deficiency may be associated with the lymphoid tissue hyperplasia characteristic of AH, potentially by downregulating systemic immune responses and increasing susceptibility to viral infections that stimulate local lymphoid hyperplasia in the Waldeyer ring (11).

Despite these findings, few studies have comprehensively evaluated the simultaneous association of vitamin D with both the allergic (AR) and hypertrophic (AH) components in the same pediatric cohort. The nature of the dose–response relationship between serum 25(OH)D levels and objective measures of disease severity, such as the adenoidal-nasopharyngeal (A/N) ratio and total IgE levels, is not well-characterized. Furthermore, the interplay of vitamin D with other risk factors like atopic predisposition has not been fully elucidated. Therefore, the present study aimed to investigate the association between serum 25(OH)D concentrations and the clinical, immunological, and radiographic severity of disease in a well-defined population of children with concurrent AR and AH. We hypothesized that lower 25(OH)D levels would be independently associated with a greater degree of adenoid hypertrophy, higher markers of allergic sensitization, and more severe clinical symptoms.

## 2 Materials and methods

### 2.1 Study design and ethical approval

This retrospective observational study was conducted at the Department of Otolaryngology-Head and Neck Surgery, Changde Hospital, Xiangya School of Medicine, Central South University (The first people’s hospital of Changde city), Hunan, China. The study protocol was approved by the hospital’s Institutional Review Board and was conducted in accordance with the Declaration of Helsinki. Written informed consent was waived due to the retrospective nature of the study, but verbal consent was obtained from parents or guardians when available.

### 2.2 Study population

We reviewed medical records of children aged 3–12 years who were diagnosed with AH combined with AR between January 2021 and December 2023. The diagnosis of AH was based on clinical symptoms (nasal obstruction, mouth breathing, snoring) and radiographic evidence with the adenoid nasopharyngeal (A/N) ratio measured on lateral nasopharyngeal radiographs (13). AR was diagnosed according to the allergic rhinitis and its impact on asthma (ARIA) guidelines, requiring at least two of the following symptoms present for more than 4 days per week and for more than 4 weeks: nasal itching, sneezing, rhinorrhea, and nasal congestion, plus positive allergen specific IgE testing (14).

#### 2.2.1 Inclusion criteria

(1) Age 3–12 years; (2) confirmed diagnosis of both AH and AR; (3) availability of serum 25(OH)D measurement within 2 weeks of initial presentation; (4) lateral nasopharyngeal radiograph with measurable A/N ratio; (5) complete allergen-specific IgE panel results; (6) no previous adenoidectomy or immunotherapy.

#### 2.2.2 Exclusion criteria

(1) Chronic diseases affecting vitamin D metabolism (chronic kidney disease, liver disease, malabsorption syndromes); (2) use of vitamin D supplements within 3 months prior to evaluation; (3) use of systemic corticosteroids within 1 month or immunosuppressive medications within 3 months; (4) presence of other chronic upper airway conditions (chronic sinusitis, nasal polyps); (5) congenital craniofacial abnormalities; (6) incomplete medical records or missing key laboratory data.

### 2.3 Sample size calculation

Sample size was calculated based on the primary objective of detecting a correlation coefficient of at least 0.25 between 25(OH)D levels and A/N ratio. Using a two-sided significance level of 0.05 and power of 90%, the minimum required sample size was calculated to be 168 subjects. To account for potential subgroup analyses and multiple comparisons, and assuming approximately 15% data exclusion due to incomplete records, we aimed to include at least 240 subjects. The sample size calculation was performed using PASS 15.0 software (NCSS, LLC, Kaysville, Utah, United States).

### 2.4 Data collection

Demographic and clinical data extracted from medical records included age, sex, body mass index (BMI), season of presentation, duration of symptoms, family history of atopy, exposure to environmental tobacco smoke (ETS), and symptom severity scores. Symptom severity was assessed using a validated pediatric rhinoconjunctivitis quality of life questionnaire with scores ranging from 0 (no symptoms) to 100 (most severe symptoms) (15).

### 2.5 Laboratory measurements

Serum 25(OH)D was measured in fasting venous blood samples (3 mL) collected between 8:00 and 10:00 a.m. Levels were quantified by electrochemiluminescence immunoassay (ECLIA) on a 25-hydroxyvitamin D analyzer (Model: AES2000A, Guangzhou Lanbo Biotechnology Co., Ltd., Guangzhou, China). Vitamin D status was categorized according to Endocrine Society criteria: severe deficiency (<10 ng/mL), deficiency (10–<20 ng/mL), insufficiency (20–<30 ng/mL), and sufficiency (≥30 ng/mL) (16).

#### 2.5.1 Immunoglobulin E measurements

Total serum IgE and specific IgE antibodies to common aeroallergens [house dust mites (Dermatophagoides pteronyssinus and Dermatophagoides farinae), cat dander, dog dander, cockroach, mold mix, tree pollen, grass pollen, and weed pollen] were measured using a fully automated protein analyzer (BN II System, Siemens, Germany). Specific IgE levels ≥0.35 kU/L were considered positive (17).

### 2.6 Radiographic assessment

Lateral nasopharyngeal radiographs were obtained in all children using standardized technique with the head in neutral position during quiet breathing (18). The A/N ratio was calculated by two independent otolaryngologists blinded to clinical and laboratory data, using the method described by Fujioka et al. (13): A/N ratio = A/N, where “A” is the distance from the point of maximal convexity of the adenoid shadow to a line along the anterior margin of the basiocciput, and “N” is the distance between the posterior border of the hard palate and the anteroinferior edge of the sphenobasioccipital synchondrosis (19). Inter-rater reliability was assessed using intraclass correlation coefficient (ICC).

### 2.7 Statistical analysis

Continuous variables were tested for normality using the Shapiro–Wilk test and presented as mean ± standard deviation (SD) for normally distributed data or median [interquartile range (IQR)] for non-normally distributed data. Categorical variables were expressed as frequencies and percentages. Comparisons among vitamin D groups were performed using one-way analysis of variance (ANOVA) with *post-hoc* Bonferroni correction for normally distributed continuous variables, Kruskal–Wallis test for non-normally distributed variables, and chi-square test or Fisher’s exact test for categorical variables.

Pearson or Spearman correlation coefficients were calculated to assess relationships between 25(OH)D levels and continuous outcomes (symptom score and specific IgE levels). Multiple linear regression models were constructed to evaluate associations between 25(OH)D levels and primary outcomes, adjusting for potential confounders including age, sex, BMI, season, duration of symptoms, environmental tobacco smoke exposure, and family history of atopy (20). Assumptions for linear and logistic regression models (e.g., linearity of logit for logistic regression, homoscedasticity, and normality of residuals for linear regression) were assessed graphically and were found to be adequately met.

To characterize the dose–response relationship between 25(OH)D levels and outcomes, restricted cubic spline (RCS) regression with three to five knots was employed (21). Knot positions were placed at recommended percentiles (10th, 50th, 90th for three knots, 5th, 35th, 65th, 95th for four knots, 5th, 27.5th, 50th, 72.5th, 95th for five knots). The optimal number of knots was determined using Akaike information criterion (AIC). Nonlinearity was tested using likelihood ratio test comparing models with and without the nonlinear spline terms.

Multivariate logistic regression analysis was performed to identify independent risk factors for severe AH (defined as A/N ratio ≥0.80) and high IgE levels (defined as total IgE >400 kU/L, based on cohort distribution and clinical significance). Variables with *p* < 0.10 in univariate analysis were included in the multivariate models. Results were presented as odds ratios (OR) with 95% confidence intervals (CI).

Subgroup analyses were conducted stratified by age (<6 years vs. ≥6 years), sex, BMI (<16.5 kg/m^2^ vs. ≥16.5 kg/m^2^), ETS exposure (Yes vs. No), family history of atopy (Yes vs. No), and season of presentation (winter vs. other seasons). Interactions were tested using multiplicative interaction terms.

A two-sided *p*-value <0.05 was considered statistically significant. All statistical analyses were performed using R software version 4.2.1 (R Foundation for Statistical Computing, Vienna, Austria).

## 3 Results

### 3.1 Study population and baseline characteristics

**Figure 1:**
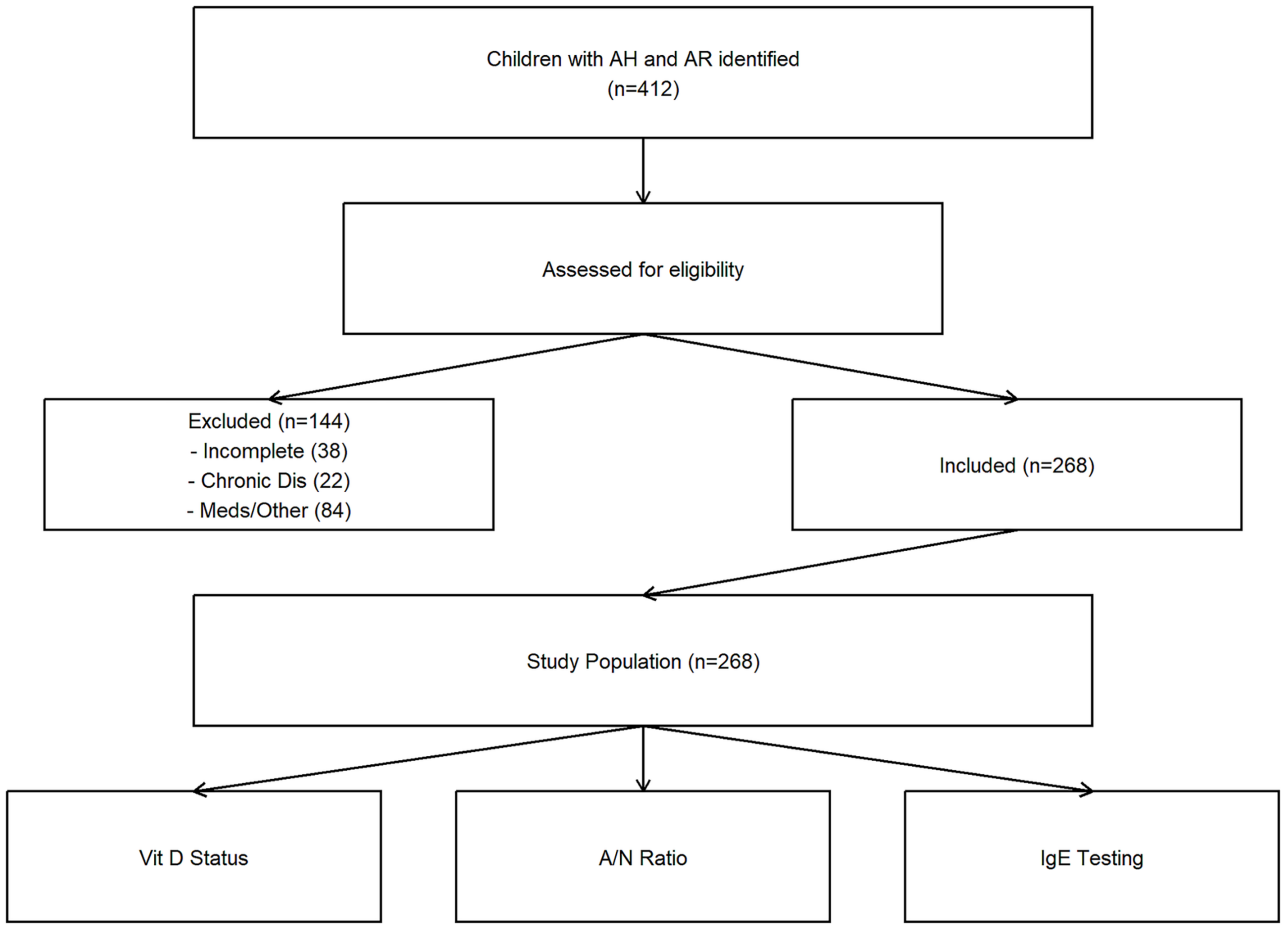
Flowchart of the study population selection process. From an initial pool of 412 potentially eligible children, 268 were included in the final analysis after applying exclusion criteria. AH, adenoid hypertrophy; AR, allergic rhinitis.

A total of 412 children with AH and AR were initially identified. After applying exclusion criteria, 268 children were included in the final analysis (Figure 1). Exclusions were due to incomplete records (*n* = 38), chronic diseases (*n* = 22), and use of disqualifying medications or other reasons (*n* = 84).

The mean age of the 268 participants was 6.9 ± 2.4 years, and 152 (56.7%) were male. The median symptom duration was 18.0 months (IQR: 12.0–22.8 months). Baseline characteristics stratified by vitamin D status are presented in Table 1.

**Table 1:** Baseline characteristics of the study population stratified by vitamin D status.

Characteristic	Severe deficiency	Deficiency	Insufficiency	Sufficiency	*p*-value
Age, years	7.6 ± 2.8	6.7 ± 2.4	7.2 ± 2.4	6.3 ± 2.0	0.087
Male sex, *n* (%)	24 (57.1)	88 (59.9)	30 (51.7)	10 (47.6)	0.595
BMI, kg/m^2^	16.7 ± 2.5	16.5 ± 1.9	16.5 ± 2.4	16.4 ± 1.8	0.928
Season of presentation, *n* (%)					<0.001
Spring	8 (19.0)	54 (36.7)	22 (37.9)	1 (4.8)	
Summer	3 (7.1)	26 (17.7)	12 (20.7)	5 (23.8)	
Autumn	6 (14.3)	30 (20.4)	14 (24.1)	11 (52.4)	
Winter	25 (59.5)	37 (25.2)	10 (17.2)	4 (19.0)	
Symptom duration, months	17.5 (13.0–27.8)	18.0 (13.0–22.5)	18.0 (11.0–21.0)	18.0 (12.0–23.0)	0.792
Family history of atopy, *n* (%)	23 (54.8)	83 (56.5)	37 (63.8)	13 (61.9)	0.741
ETS exposure, *n* (%)	16 (38.1)	54 (36.7)	19 (32.8)	9 (42.9)	0.858
25(OH)D, ng/mL	7.5 ± 1.8	15.3 ± 2.6	25.4 ± 2.7	33.6 ± 3.0	<0.001
A/N ratio	0.92 ± 0.09	0.82 ± 0.12	0.79 ± 0.12	0.76 ± 0.13	<0.001
Total IgE, kU/L	531 (336–798)	325 (192–506)	224 (126–492)	248 (146–368)	<0.001
Symptom score	79.3 ± 11.7	67.0 ± 13.1	56.1 ± 12.6	57.1 ± 10.6	<0.001

Data are presented as mean ± SD, median (IQR), or *n* (%). *p*-values are from ANOVA for normal continuous data, Kruskal–Wallis for non-normal continuous data, and chi-square test for categorical data. BMI, body mass index; ETS, environmental tobacco smoke; 25(OH)D, 25-hydroxyvitamin D; A/N ratio, adenoid-nasopharynx ratio; IgE, immunoglobulin E.

The overall mean serum 25(OH)D level was 17.7 ± 6.8 ng/mL. A high prevalence of vitamin D deficiency was observed: 42 children (15.7%) had severe deficiency (<10 ng/mL), 147 (54.9%) had deficiency (10–<20 ng/mL), 58 (21.6%) had insufficiency (20–<30 ng/mL), and only 21 (7.8%) had sufficient levels (≥30 ng/mL). Thus, 189 children (70.5%) were vitamin D deficient (<20 ng/mL). As expected, 25(OH)D levels varied significantly by season (*p* < 0.001), with the highest proportion of severe deficiency occurring in winter (59.5% of the severely deficient group presented in winter).

The overall mean A/N ratio was 0.82 ± 0.12, and the overall median total IgE level was 306 kU/L (IQR: 178–501 kU/L). Inter-rater reliability for A/N ratio measurements showed excellent agreement (ICC = 0.94, 95% CI: 0.92–0.96). As shown in Table 1, there was a clear gradient of disease severity across vitamin D groups. Children with severe deficiency had significantly higher mean A/N ratios (0.92 ± 0.09), median total IgE levels (531 kU/L), and symptom scores (79.3 ± 11.7) compared to all other groups (all *p* < 0.001).

### 3.2 Correlations between 25(OH)D and disease severity markers

**Figure 2:**
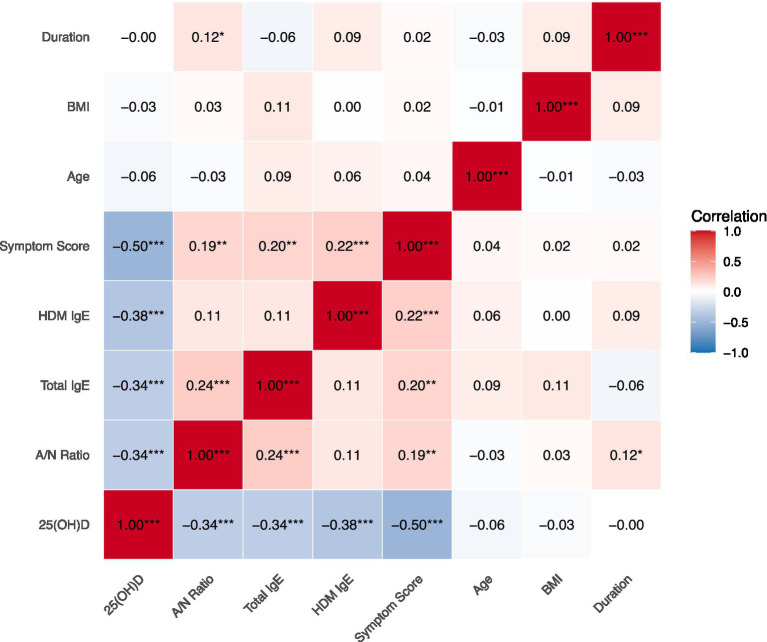
Correlation matrix of serum 25(OH)D levels and disease severity markers. The color intensity and size of the circles are proportional to the correlation coefficients. ^*^p < 0.05, ^**^p < 0.01, and ^***^p < 0. 001.

As depicted in the correlation matrix (Figure 2), significant inverse correlations were observed between serum 25(OH)D levels and all primary outcome measures. 25(OH)D showed a strong negative correlation with symptom scores (*r* = −0.50, *p* < 0.001) and moderate negative correlations with A/N ratio (*r* = −0.34, *p* < 0.001), total IgE (*r* = −0.34, *p* < 0.001), and HDM-specific IgE (*r* = −0.38, *p* < 0.001). Weak positive correlations were noted between the severity markers themselves, such as between A/N ratio and total IgE (*r* = 0.24, *p* < 0.001).

### 3.3 Comparisons across vitamin D status groups

Disease severity indicators showed a stark, dose-dependent relationship with vitamin D status (Table 2). The proportion of children with severe AH (A/N ratio ≥0.80) progressively increased from 42.9% in the sufficiency group to 83.3% in the severe deficiency group (*p* for trend = 0.001). A similar, even more pronounced trend was seen for a stricter cutoff of A/N ratio ≥0.85 (23.8% vs. 81.0%, *p* for trend <0.001).

**Table 2:** Disease severity indicators according to vitamin D status.

Outcome	Severe deficiency	Deficiency	Insufficiency	Sufficiency	*p* for trend
A/N ratio ≥0.80, *n* (%)	35 (83.3)	81 (55.1)	28 (48.3)	9 (42.9)	<0.001
A/N ratio ≥0.85, *n* (%)	34 (81.0)	70 (47.6)	17 (29.3)	5 (23.8)	<0.001
Total IgE >400 kU/L, *n* (%)	28 (66.7)	59 (40.1)	16 (27.6)	5 (23.8)	<0.001
Polysensitization (≥3), *n* (%)	34 (81.0)	74 (50.3)	16 (27.6)	2 (9.5)	<0.001
Moderate-to-severe symptoms, *n* (%)	38 (90.5)	105 (71.4)	24 (41.4)	6 (28.6)	<0.001

Data are presented as *n* (%). *p*-values are from chi-square test. *p* for trend is from the Cochran–Armitage trend test. A/N ratio, adenoid-nasopharynx ratio; IgE, immunoglobulin E; HDM, house dust mite. Moderate–severe symptoms defined as symptom score ≥60.

Likewise, the prevalence of high total IgE levels increased as vitamin D status declined. The proportion of children with total IgE >400 kU/L was nearly three times higher in the severe deficiency group (66.7%) compared to the sufficiency group (23.8%, *p* for trend <0.001). The prevalence of polysensitization (≥3 positive allergens) and moderate-to-severe symptoms also followed this significant inverse trend (both *p* for trend <0.001).

### 3.4 Dose–response relationships

**Figure 3:**
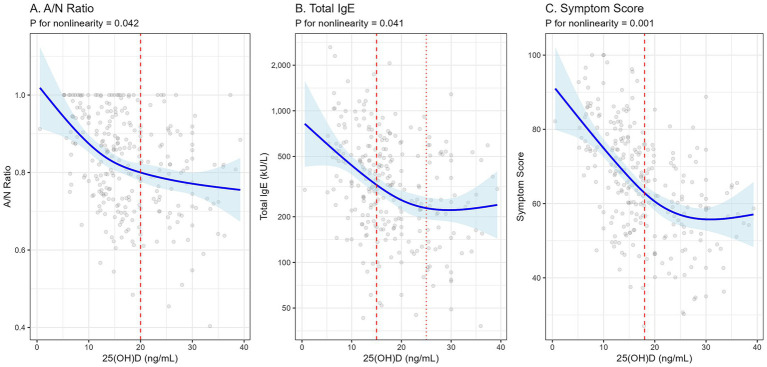
Nonlinear dose–response relationship between serum 25(OH)D levels and disease severity. Restricted cubic spline regression models showing the adjusted association between continuous 25(OH)D levels and **(A)** A/N ratio, **(B)** total IgE, and **(C)** symptom scores. The solid line represents the adjusted mean, and the shaded area represents the 95% confidence interval. Models were adjusted for age, sex, BMI, season, symptom duration, ETS exposure, and family history of atopy.

Restricted cubic spline regression analysis confirmed significant nonlinear dose–response relationships between 25(OH)D levels and A/N ratio (*p* for nonlinearity = 0.042), total IgE (*p* for nonlinearity = 0.041), and symptom scores (*p* for nonlinearity = 0.001) (Figure 3). For all three outcomes, the curves demonstrated a steep inverse association at lower 25(OH)D levels, particularly below 20–25 ng/mL, which then flattened out at higher concentrations. This indicates that improvements in disease markers are most pronounced when moving from a deficient to an insufficient state, suggesting a potential threshold effect. The relationship between 25(OH)D and A/N ratio, for example, showed a sharp decline until approximately 25 ng/mL, after which the curve became much more gradual.

### 3.5 Multiple linear regression analysis

After adjusting for age, sex, BMI, season, symptom duration, ETS exposure, and family history of atopy, 25(OH)D remained a powerful and independent predictor of disease severity (Table 3). Each 10 ng/mL decrease in continuous 25(OH)D was associated with a 0.055-unit increase in A/N ratio (*p* < 0.001), a 130 kU/L increase in total IgE (*p* < 0.001), and a 9.78-point increase in symptom score (*p* < 0.001).

**Table 3:** Multiple linear regression analysis for factors associated with A/N ratio, total IgE, and symptom score.

Variable	Adjusted *β* (95% CI) (A/N ratio)	*p*-value	Adjusted *β* (95% CI) (total IgE)	*p*-value	Adjusted *β* (95% CI) (symptom score)	*p*-value
25(OH)D (per 10 ng/mL decrease)	0.055 (0.036, 0.074)	<0.001	130 (77, 184)	<0.001	9.78 (7.67, 11.89)	<0.001
25(OH)D categories (ref: ≥30 ng/mL)						
<10 ng/mL	0.169 (0.105, 0.233)	<0.001	366 (186, 546)	<0.001	23.0 (15.9, 30.0)	<0.001
10–<20 ng/mL	0.076 (0.021, 0.131)	0.007	152 (−2, 307)	0.053	10.2 (4.1, 16.2)	0.001
20–<30 ng/mL	0.043 (−0.016, 0.103)	0.154	38 (−129, 206)	0.653	−1.0 (−7.6, 5.6)	0.765
Age	−0.003 (−0.009, 0.003)	0.303	4 (−12, 20)	0.634	0.04 (−0.60, 0.68)	0.908
Male sex	−0.025 (−0.054, 0.003)	0.084	−25 (−106, 55)	0.536	−1.2 (−4.4, 1.9)	0.442
BMI	0.000 (−0.007, 0.006)	0.936	22 (4, 41)	0.020	0.01 (−0.72, 0.73)	0.987
Winter season	0.032 (−0.005, 0.069)	0.086	145 (42, 248)	0.006	−0.08 (−4.1, 4.0)	0.971
Symptom duration	0.002 (0.001, 0.004)	0.010	−4 (−9, 1)	0.136	0.06 (−0.13, 0.25)	0.535
ETS exposure	0.016 (−0.013, 0.045)	0.265	−24 (−106, 57)	0.557	1.9 (−1.3, 5.1)	0.240
Family history of atopy	0.028 (0.000, 0.057)	0.052	110 (30, 190)	0.007	3.0 (−0.2, 6.2)	0.065

Models adjusted for all variables shown. The first row for 25(OH)D shows the effect of a 10 ng/mL decrease in continuous 25(OH)D levels. A/N, adenoid-nasopharynx; IgE, immunoglobulin E; BMI, body mass index; ETS, environmental tobacco smoke; CI, confidence interval. Total IgE was log-transformed for analysis; betas are back-transformed for interpretability.

When 25(OH)D was analyzed as a categorical variable (with sufficiency as reference), severe deficiency (<10 ng/mL) was associated with a 0.169-unit higher A/N ratio (*p* < 0.001), a 366 kU/L higher total IgE level (*p* < 0.001), and a 23.0-point higher symptom score (*p* < 0.001) after full adjustment. Other significant predictors for worse outcomes included winter season presentation, longer symptom duration, and a positive family history of atopy.

### 3.6 Risk factors for severe disease

Multivariate logistic regression analysis reinforced the critical role of vitamin D status in severe disease (Table 4). Compared to children with sufficient vitamin D, those with severe deficiency (<10 ng/mL) had a dramatically increased risk of severe AH (A/N ratio ≥0.80), with an odds ratio of 7.13 (95% CI: 2.06–26.73, *p* = 0.002). For the moderate deficiency group (10–<20 ng/mL), the point estimate for the odds ratio was elevated (OR = 1.78), but the 95% confidence interval was wide and included 1.0 (*p* = 0.252), indicating no statistically significant association with severe AH in this subgroup. Exposure to environmental tobacco smoke was also identified as an independent risk factor for severe AH (OR = 1.76, 95% CI: 1.03–3.05, *p* = 0.041).

**Table 4:** Multivariate logistic regression analysis for risk factors for severe AH and high total IgE.

Variable	Odds ratio (95% CI) (severe AH (A/N ratio ≥0.80))	*p*-value	Odds ratio (95% CI) (high total IgE (>400 kU/L))	*p*-value
25(OH)D categories (ref: ≥30 ng/mL)				
<10 ng/mL	7.13 (2.06, 26.73)	0.002	7.83 (1.90, 37.37)	0.006
10–<20 ng/mL	1.78 (0.67, 4.87)	0.252	2.38 (0.82, 6.76)	0.104
20–<30 ng/mL	1.38 (0.48, 4.09)	0.555	1.06 (0.34, 3.22)	0.921
Age	0.94 (0.84, 1.04)	0.243	1.03 (0.91, 1.17)	0.597
Male sex	0.68 (0.40, 1.15)	0.153	1.27 (0.70, 2.29)	0.435
Winter season	1.43 (0.71, 2.90)	0.318	1.49 (0.68, 3.33)	0.326
Symptom duration	1.02 (0.99, 1.06)	0.176	1.02 (0.98, 1.06)	0.429
ETS exposure	1.76 (1.03, 3.05)	0.041	1.02 (0.56, 1.89)	0.941
Family history of atopy	1.61 (0.96, 2.74)	0.075	3.74 (2.09, 6.85)	<0.001

Models adjusted for all variables shown plus BMI. AH, adenoid hypertrophy; A/N, adenoid-nasopharynx ratio; IgE, immunoglobulin E; 25(OH)D, 25-hydroxyvitamin D; ETS, environmental tobacco smoke; OR, odds ratio; CI, confidence interval.

For high total IgE (>400 kU/L), severe vitamin D deficiency was an even stronger predictor, with an odds ratio of 7.83 (95% CI: 1.90–37.37, *p* = 0.006). A positive family history of atopy was also a major risk factor for high IgE levels (OR = 3.74, 95% CI: 2.09–6.85, *p* < 0.001).

### 3.7 Allergen-specific IgE patterns

**Figure 4:**
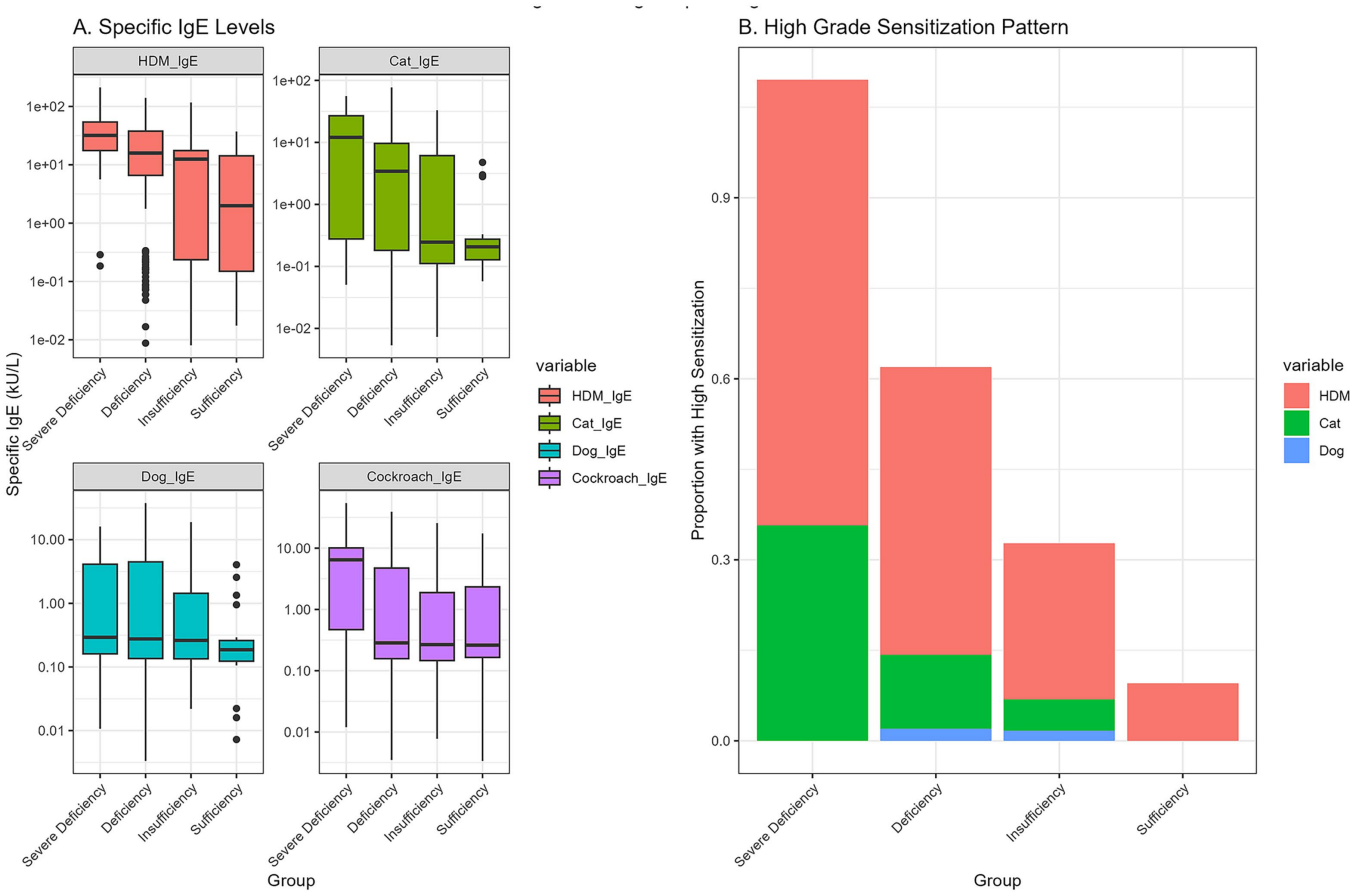
Allergen-specific IgE sensitization patterns by vitamin D status. Boxplots showing the median and interquartile range of specific IgE levels for major allergens across the four vitamin D status groups.

Analysis of allergen sensitization revealed that lower vitamin D status was associated with both a broader and more intense allergic response (Table 5 and Figure 4). The median number of positive allergens was significantly higher in the severe deficiency group (median: 3, IQR: 3–4) compared to the sufficiency group (median: 1, IQR: 1–2, *p* < 0.001).

**Table 5:** Allergen-specific IgE patterns according to vitamin D status.

Allergen	Severe deficiency	Deficiency	Insufficiency	Sufficiency	*p*-value
House dust mites, positive, *n* (%)	40 (95.2)	119 (81.0)	37 (63.8)	11 (52.4)	<0.001
House dust mites, specific IgE, kU/L	33.2 (19.7–54.4)	21.9 (12.1–43.3)	16.3 (13.0–26.0)	14.2 (7.3–16.8)	0.001
Cat dander, positive, *n* (%)	28 (66.7)	81 (55.1)	23 (39.7)	3 (14.3)	<0.001
Cat dander, specific IgE, kU/L	24.1 (12.1–31.8)	8.9 (5.6–15.1)	7.4 (5.2–12.5)	3.0 (2.9–3.9)	<0.001
Dog dander, positive, *n* (%)	18 (42.9)	54 (36.7)	17 (29.3)	4 (19.0)	0.211
Dog dander, specific IgE, kU/L	4.9 (3.7–7.0)	6.2 (4.1–10.8)	4.2 (2.6–6.9)	2.0 (1.2–2.9)	0.015
Cockroach, positive, *n* (%)	31 (73.8)	58 (39.5)	17 (29.3)	6 (28.6)	<0.001
Cockroach, specific IgE, kU/L	8.4 (5.4–12.5)	7.9 (3.5–15.5)	5.8 (2.7–9.3)	4.0 (3.1–5.6)	0.151
Tree pollen, positive, *n* (%)	21 (50.0)	55 (37.4)	15 (25.9)	3 (14.3)	0.014
Tree pollen, specific IgE, kU/L	4.2 (3.6–10.7)	3.9 (2.1–7.5)	3.6 (2.1–5.0)	2.7 (1.7–3.8)	0.149
Number of positive allergens, median (IQR)	3 (3–4)	3 (2–3)	2 (1–3)	1 (1–2)	<0.001

Data presented as *n* (%) for positive rates (specific IgE ≥0.35 kU/L) or median (IQR) for specific IgE levels and number of positive allergens. *p*-values are from chi-square/Fisher’s exact test for rates and Kruskal–Wallis test for medians.

For house dust mites (HDM), the most prevalent allergen, both the positivity rate (95.2% vs. 52.4%, *p* < 0.001) and the median specific IgE level (33.2 vs. 14.2 kU/L, *p* = 0.001) were significantly worse in the severe deficiency group compared to the sufficiency group. Similar significant trends were observed for sensitization to cat dander, dog dander, cockroach, and tree pollen, demonstrating a systemic effect of vitamin D on allergic sensitization.

### 3.8 Subgroup and sensitivity analyses

**Figure 5:**
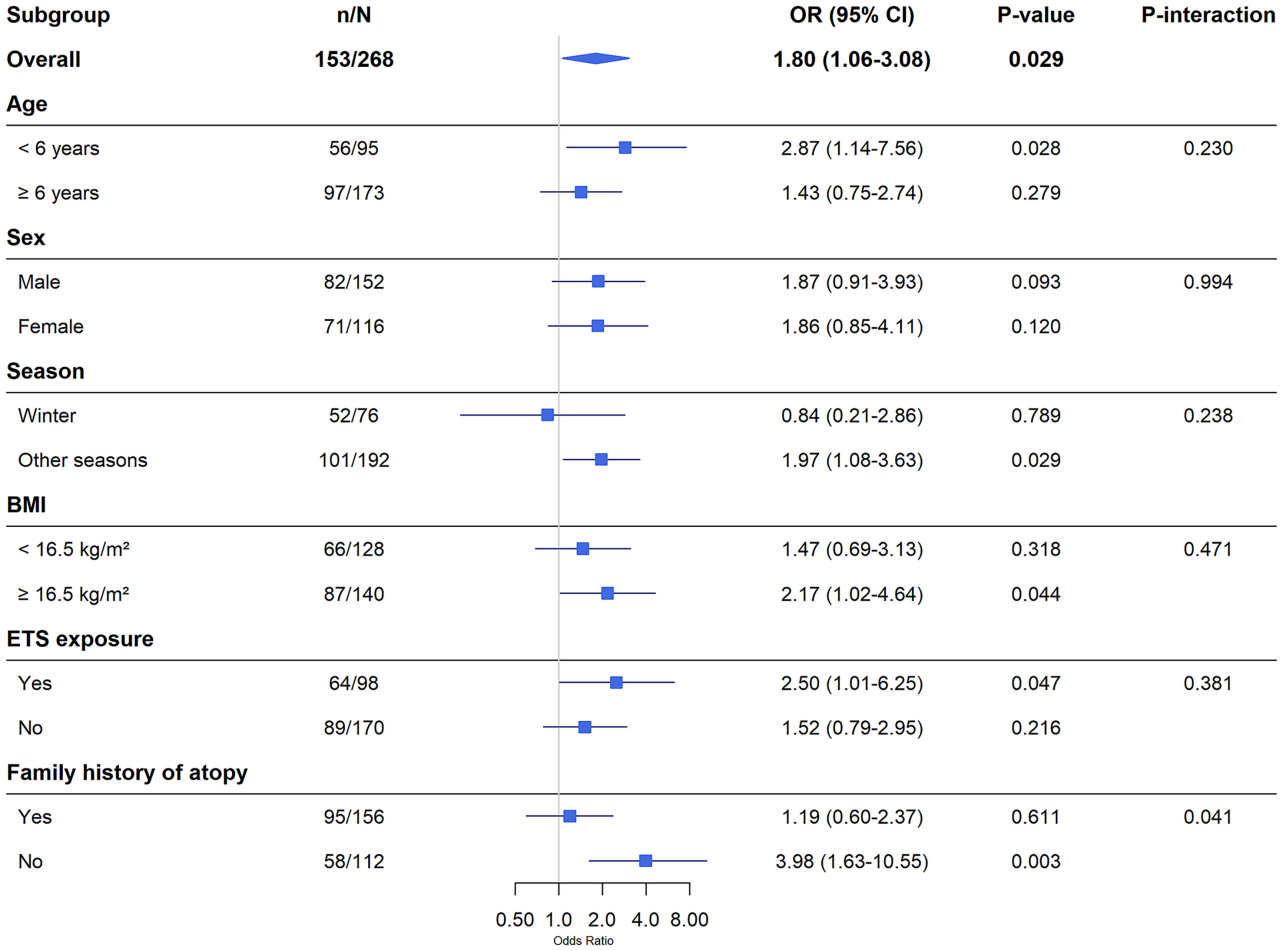
Subgroup analysis of the association between vitamin D deficiency and severe AH. Forest plot showing the odds ratios for severe AH (A/N ratio ≥0.80) associated with vitamin D deficiency (<20 ng/mL) across different subgroups.

Subgroup analyses were performed to assess the association between vitamin D deficiency (<20 ng/mL) and severe AH (A/N ratio ≥0.80) across different strata (Figure 5). The analysis revealed a significant interaction between vitamin D status and family history of atopy (*p*-interaction = 0.041). The odds ratio for severe AH associated with vitamin D deficiency was substantially higher in children with a positive family history of atopy (OR = 3.98, 95% CI: 1.63–10.55, *p* = 0.003) compared to those without (OR = 1.19, 95% CI: 0.60–2.37, *p* = 0.611). This suggests that children with an atopic predisposition are particularly vulnerable to the effects of vitamin D deficiency on adenoid growth. No other significant interactions were detected for age, sex, season, BMI, or ETS exposure, indicating that the association was otherwise consistent across these subgroups.

## 4 Discussion

This study reveals a compelling and clinically significant association between vitamin D deficiency and the severity of co-existing allergic rhinitis and adenoid hypertrophy in children. The principal findings demonstrate that an overwhelming majority of these children—over 70%—were deficient in vitamin D, and this deficiency was not merely a coincidental finding. Lower serum 25(OH)D levels were independently and robustly associated with greater adenoid size, as measured by the A/N ratio; higher levels of allergic sensitization, reflected by total and allergen-specific IgE; and worse clinical symptom scores. The strength of these associations persisted even after adjusting for a host of potential confounding factors, underscoring the potential correlation of vitamin D status with disease severity in this common pediatric comorbidity.

A particularly insightful finding of our investigation is the nonlinear dose–response relationship observed between 25(OH)D levels and the primary outcomes, as depicted by the restricted cubic spline analysis. For A/N ratio, total IgE, and symptom scores, the curves were steepest at the lower end of the vitamin D spectrum, specifically below levels of 20–25 ng/mL. This suggests a potential threshold effect, where the strongest association with detrimental outcomes on airway inflammation and lymphoid tissue growth is most pronounced. The subsequent flattening of the curves at higher concentrations implies that the greatest clinical benefit may be derived from correcting outright deficiency and elevating serum levels to at least an insufficient or, ideally, sufficient state. This finding has direct clinical implications, suggesting that intervention efforts should prioritize patients with the most severe deficiency, as they stand to gain the most significant improvement. This dose–response pattern aligns with the biological understanding of hormone-receptor interactions, where cellular responses may become saturated once a certain concentration of the ligand—in this case, 1,25(OH)2D3—is achieved.

The observed associations are biologically plausible and are strongly supported by the well-documented immunomodulatory functions of vitamin D. Allergic rhinitis is a classic Th2-mediated disease, and our finding that lower vitamin D levels are linked with higher total IgE, a higher number of positive allergens, and greater specific IgE responses to common aeroallergens like house dust mites, supports the hypothesis that vitamin D deficiency may contribute to fostering a pro-allergic immune environment. Vitamin D modulates Th2 responses, including impairing IL-4 secretion, which is critical for B-cell class switching to IgE production, and by promoting the function of regulatory T cells (22, 23). A deficiency could plausibly disinhibit this pathway, leading to the exaggerated allergic sensitization observed in our cohort. Simultaneously, the link between low vitamin D and increased adenoid size points to its role in regulating lymphoid tissue homeostasis. Adenoids are immunologically active tissues, and their hypertrophy is often driven by chronic inflammation and recurrent infections, which are key drivers. Vitamin D contributes to innate mucosal defense, in part by supporting the production of antimicrobial peptides like LL-37, and helps maintain epithelial barrier integrity (23, 24). Consequently, its deficiency could lead to increased microbial colonization and a state of chronic, low-grade inflammation in the nasopharynx, and is associated with an increased risk of recurrent tonsillitis, a condition characterized by lymphoid tissue hyperplasia (25, 26). Our results, which tie a single factor—vitamin D deficiency—to both the allergic (IgE) and hypertrophic (A/N ratio) arms of this comorbidity, provide strong clinical evidence for an association with both arms of this comorbidity, suggesting a potential unified pathogenic mechanism.

The findings from our multiple regression analyses, which identified severe vitamin D deficiency as a powerful independent risk factor for both severe AH and high total IgE, are consistent with previous reports. For example, De Luca et al. (11) reported significantly lower vitamin D levels in children with adenotonsillar hypertrophy and OSA, and Shin et al. (12) found an inverse correlation between vitamin D levels and adenotonsillar size. Our study extends these findings by using robust statistical modeling in a larger, well-characterized cohort of children with confirmed AR, providing more precise effect estimates and establishing a clear dose–response relationship that previous studies have not fully detailed. Furthermore, the identification of environmental tobacco smoke exposure as another independent risk factor for severe AH aligns with its known role as a respiratory irritant that can promote mucosal inflammation.

One of the most novel and important findings of our study is the significant interaction between vitamin D status and a family history of atopy. Vitamin D deficiency had a much stronger effect on the risk of severe AH in children with an atopic family background. This may represent a classic gene–environment interaction. It is plausible that children with a genetic predisposition to atopy may have an underlying immune system that is more sensitive to the dysregulating effects of vitamin D deficiency. Their immune cells might exhibit a more pronounced shift towards a Th2 phenotype or have a more limited capacity to induce regulatory responses in the absence of sufficient vitamin D signaling. This suggests that children with atopy in their families are a particularly high-risk subgroup that should be prioritized for vitamin D status screening and potential intervention.

The strengths of this study include a relatively large sample size, the use of standardized and objective measures for both AH (A/N ratio) and allergic sensitization (ImmunoCAP), and the application of sophisticated statistical methods, including restricted cubic splines and interaction analysis, to explore the nuances of the observed associations. However, several limitations must be acknowledged. First, the retrospective, observational design precludes the establishment of causality; we can demonstrate a strong association, but not prove that vitamin D deficiency causes the worsening of AR and AH. It is also possible that severe chronic disease could lead to behavioral changes (e.g., less sun exposure) that lower vitamin D levels, representing a degree of reverse causation. Secondly, being a single-center study, our findings may not be generalizable to other populations with different genetic backgrounds, dietary habits, or levels of sun exposure. The study cohort was recruited from a single tertiary hospital, which likely resulted in a selection bias towards children with more severe or refractory symptoms who were referred for specialist care. Therefore, our findings, particularly the high prevalence of vitamin D deficiency, may not be generalizable to the general pediatric population or to children with milder forms of AR and AH. Third, although we adjusted for several key confounders, the possibility of residual confounding by unmeasured variables cannot be entirely ruled out. Our study is limited by the lack of data on certain lifestyle factors known to influence vitamin D levels, such as specific dietary habits, time spent outdoors, physical activity, and socioeconomic status. For instance, underlying systemic inflammation, which we did not directly measure, could independently influence both vitamin D metabolism and the severity of atopic conditions, thus acting as a residual confounder. Furthermore, vitamin D status was assessed via a single serum measurement, which reflects recent but not necessarily long-term status, though this is a common approach in clinical and epidemiological studies. Finally, our analysis involved multiple comparisons, and we did not apply a formal correction like the Bonferroni method. However, our primary findings regarding the association between severe vitamin D deficiency and key outcomes were highly significant (*p* < 0.001), suggesting they would remain robust even after such an adjustment.

In conclusion, this hypothesis-generating study provides strong evidence that vitamin D deficiency is independently and quantitatively associated with the severity of both adenoid hypertrophy and allergic rhinitis in children. The relationship is dose-dependent and is particularly influential in children with a family history of atopy. These findings have significant clinical implications, suggesting that the assessment and correction of vitamin D status could represent a simple, cost-effective, and safe adjuvant strategy in the management of this challenging pediatric comorbidity. While awaiting definitive evidence from large-scale, prospective randomized controlled trials to confirm the causal and therapeutic benefit of supplementation, clinicians should consider screening for vitamin D deficiency in children presenting with significant nasal obstruction and allergic symptoms, especially in those who are refractory to standard therapies.

## Data Availability

The raw data supporting the conclusions of this article will be made available by the authors, without undue reservation.

## Glossary

A/N: Adenoidal-nasopharyngeal
AH: Adenoid hypertrophy
AIC: Akaike information criterion
ANOVA: Analysis of variance
AR: Allergic rhinitis
ARIA: Allergic rhinitis and its impact on asthma
BMI: Body mass index
Breg: Regulatory B cell
CI: Confidence interval
ECLIA: Electrochemiluminescence immunoassay
ETS: Environmental tobacco smoke
HDM: House dust mites
ICC: Intraclass correlation coefficient
IgE: Immunoglobulin E
IL: Interleukin
IQR: Interquartile range
OR: Odds ratio
OSA: Obstructive sleep apnea
RCS: Restricted cubic spline
SDB: Sleep-disordered breathing
SD: Standard deviation
Tregs: Regulatory T cells
VDR: Vitamin D receptor
25(OH)D: 25-hydroxyvitamin D
1,25(OH)2D3: 1,25-dihydroxyvitamin D3
